# Brain metabolism and related connectivity in patients with acrophobia treated by virtual reality therapy: an ^18^F-FDG PET pilot study sensitized by virtual exposure

**DOI:** 10.1186/s13550-018-0446-9

**Published:** 2018-10-01

**Authors:** Antoine Verger, Eric Malbos, Emmanuelle Reynaud, Pierre Mallet, Daniel Mestre, Jean-Marie Pergandi, Stéphanie Khalfa, Eric Guedj

**Affiliations:** 1 Department of Nuclear Medicine and Nancyclotep Imaging platform, CHRU Nancy, Lorraine University, Nancy, France; 2 IADI, INSERM, UMR 1254, Lorraine University, Nancy, France; 3 Department of Psychiatry, La Conception University Hospital, Marseille, France; 4 Aix-Marseille Université, CNRS, Ecole Centrale Marseille, UMR 7249, Institut Fresnel, Marseille, France; 5 Institute of Neurosciences, CNRS UMR7289, Aix-Marseille Université, Timone University Hospital, Marseille, France; 6 Aix-Marseille Université, CNRS, ISM, Institute of Movement Sciences, Marseille, France; 7 Department of Nuclear Medicine, Assistance Publique-Hôpitaux de Marseille, Timone University Hospital, Marseille, France; 8 CERIMED, Aix-Marseille Université, Marseille, France

**Keywords:** Acrophobia, Virtual reality exposure therapy, PET, Metabolic connectivity

## Abstract

**Background:**

The aim of this pilot study is to investigate the impact of virtual reality exposure therapy (VRET) on brain metabolism and connectivity.

Eighteen patients with acrophobia were assessed by an ^18^F-FDG PET scan sensitized by virtual exposure before treatment, and nine of them were assessed again after eight sessions of VRET. Statistical Parametric Mapping was used to study the correlations between metabolism and pretherapeutic clinical scores and to compare metabolism before and after VRET (*p* voxel < 0.005, corrected for cluster volume). Metabolic connectivity was evaluated through interregional correlation analysis.

**Results:**

Before therapy, a positive correlation was found between scores on the behavioural avoidance test and left occipital metabolism (BA17-18). After VRET, patients presented increased metabolism in the left frontal superior gyri and the left precentral gyrus, which showed increased metabolic connectivity with bilateral occipital areas (BA17-18-19), concomitant with clinical recovery.

**Conclusions:**

This study highlights the exciting opportunity to use brain PET imaging to investigate metabolism during virtual exposure and reports the involvement of the visual-motor control system in the treatment of acrophobia by VRET.

## Background

Acrophobia, defined as “fear of heights”, is a specific phobia according to the criteria of the “Diagnostic and statistical manual of mental disorders” (DSM-5) [1, 2], with high prevalence, chronicity and cumulative social impact [3].

For the past two decades, virtual reality exposure therapy (VRET) has been proposed to treat acrophobia. The principle is to immerse the patient in a computer-generated virtual environment [4]. VRET has the advantage of conducting time-consuming exposure therapy without the need of getting outside medical institutions. Interestingly, in acrophobia, VRET was found to be at least as effective as in vivo exposure for anxiety and avoidance [5], with clinical beneficial effects maintained at least 1 year after treatment [6]. VRET also provides the unique opportunity to study pathophysiological changes in an ecologically relevant setting during phobic exposure. However, until now, no functional brain imaging technique has been used to objectify the VRET response.

PET imaging provides the competitive advantage of allowing ^18^F-FDG administration outside the imaging device to study metabolic changes during virtual exposure. Beyond the identification of metabolic dysfunction within individual brain regions, the analysis of metabolic connectivity using inter-regional correlation analysis (IRCA) leads to a better understanding on the network scale [7].

The aim of this ^18^F-FDG PET pilot study, sensitized by virtual exposure during radiopharmaceutical administration, was to investigate the impact of VRET on brain metabolism and related connectivity.

## Methods

### Selection of patients, controls and study design

From May 2015 to September 2016, 18 patients (46 ± 11 years old, five women) diagnosed with acrophobia were enrolled in the prospective CTRLSTRESS study (NCT02020824) approved by the Institutional Review Board CPP-Sud Méditerranée (2013-A01280-45). Population characteristics are available in Table 1. At inclusion, right-handed patients must be between 18 and 60 years old and suffer from acrophobia according to DSM-5 criteria [2]. Exclusion criteria comprised psychotropic pharmacotherapy for a period of less than 8 weeks for treatment stabilization and behavioural avoidance test (BAT) score superior to 6. Similarly, patients continuing psychotherapy, suffering from neurological disorders or comorbid psychiatric diseases other than acrophobia, or suffering from severe organic disorders that could disable or disrupt the therapeutic process were also not included. Patients were assessed by a ^18^F-FDG PET scan, sensitized by virtual exposure during the radiopharmaceutical administration, before VRET and 2 months later after eight therapeutic VR sessions (1-h sessions separated from each other by 9 days). Psychological assessments included the subjective unit of discomfort (SUD) test [8] on a 100-point scale, performed immediately after virtual exposure of the pre- and post-therapeutic ^18^F-FDG PET scan, and an objective behavioural instrument such as the BAT. The BAT is a direct behavioural observation of distress in response to entering a feared situation [9]. It was measured by observing the subject’s performance in a specific height-related virtual environment comprising a walk over a transparent platform located above an 800-m (2640 ft) canyon. The score on a 10-point scale relies on the distance covered by the subject while immersed in this virtual environment and was calculated before and after VRET [10]. BAT and SUD scores’ characteristics are detailed in Table 2. All patients participated with informed written consent in accordance with the Declaration of Helsinki.

**Table 1:** Characteristics of the patients

	All patients *n* = 18	Patients with ^18^F-FDG PET before and after treatment *n* = 9
Age, years	46.0 (SD = 11.0)	47.4 (SD = 11.6)
Gender: female	5 (28%)	4 (44%)
Laterality (right-handed)	13 (72%)	6 (66%)
Duration of acrophobia, years	3.0 (SD = 7.1)	1.2 (SD = 0.4)
Video game experience	10 (56%)	4 (44%)
Psychometric assessment		
BAT (/10) before treatment	3.7 (SD = 1.1)	4.6 (SD = 2.2)
SUD (/100) before treatment	48.3 (SD = 27.1)	42.8 (SD = 33.3)
BAT (/10) after treatment	–	8.8 (SD = 1.8)*
SUD (/100) after treatment	–	8.9 (SD = 20.3)*

*BAT* behavioural avoidance test, *SUD* subjective unit of discomfort

*p < 0.05 for comparison of results before and after treatment

**Table 2:** BAT and SUD scores’ characteristics

	BAT score	SUD score
Definition	Behavioural avoidance test	Subjective unit of discomfort
Scale	/10	/100
Interpretation	A low score is in favour of avoidance	A high score is in favour of discomfort
Test	This test is specific to this study. The subject has to walk, in a virtual environment, over a transparent platform located above an 800-m (2640 ft) canyon.	The subject has to determine his/her own feeling of discomfort.
Scoring	The score on a 10-point scale relies on the distance covered by the subject while immersed in the virtual environment.	The score is on a 100-point scale. The subject is his/her own reference.
Estimated healthy controls score	10	0

### ^18^F-FDG PET acquisition and analysis

**Fig. 1:**
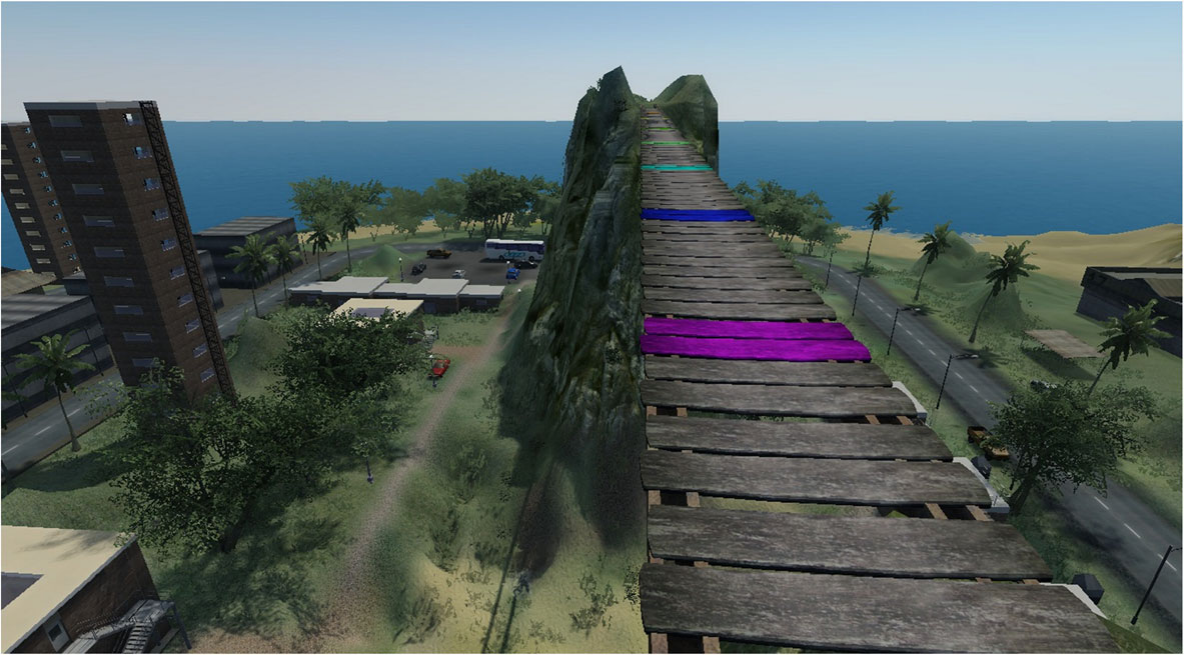
Screen capture of the virtual environment created for the ^18^F-FDG PET scan sensitized by virtual exposure consisting of a 50-m (165 ft) high wooden bridge devoid of any handrails and made of spaced planks. Coloured planks are dependent on the distance covered by the subject while immersed in this virtual environment for calculating the behavioural avoidance test (BAT) score on a 10-point scale

^18^F-FDG PET was performed under the same conditions for all patients, using an integrated PET/CT camera (Discovery ST, GE Healthcare, Waukesha, WI) with an axial resolution of 6.2 mm allowing 47 contiguous transverse sections of the brain of 3.27 mm thickness. ^18^F-FDG (150 MBq) was injected intravenously while the subjects were standing awake and sensitized by virtual exposure: the subject was asked to don a Sensics zSight™ head-mounted display (HMD, 800 × 600 stereoscopic OLED screen with 60° field of view) coupled with a VirtualCube™, i.e. a 3 degrees of freedom motion head tracker (angular resolution: 0.02°, latency 4 ms). Once linked to the computer main unit and associated with the head tracker, the HMD displays images which are updated in real time depending on the user’s head spatial orientation. The subject was standing up in a 4-m^2^ area and being confronted with intense phobia cues, consisting of a 50-m (165 ft) high wooden bridge devoid of any handrails and made of spaced planks, as described in Fig. 1. Patients could navigate freely through the virtual environment with a Mobility Lab™ navigational controller in one of their hands allowing virtual locomotion (walking forward and backward). The controller was required to be lightweight for extended intervention, wireless, to be handled with a single hand for comfort, to feature a directional pad for easy navigation and with additional buttons for specific functions (e.g. interact and sit). Therefore, orientation in the virtual environment was possible through physical rotation of the head and body as well as the use of the directional pad for virtual locomotion in the chosen direction. This virtual environment was created specifically for the ^18^F-FDG PET scan and was distinct from the ones exploited for the eight therapeutic VR sessions in the cave automatic virtual environment (CAVE). After an explanation of the principle of the test, patient was immerged in the virtual environment. At the eighth minute after the beginning of the immersion in the virtual environment, ^18^F-FDG was injected, and the virtual exposure immersion was maintained after radiopharmaceutical injection for approximately 7 min. While the administration of ^18^F-FDG was performed, the subject was proposed although never coerced to walk on the virtual bridge as far as he could. The subjective unit of discomfort (SUD) test was performed immediately after VR exposure of the pre- and post-therapeutic ^18^F-FDG PET scan, consisting of only one question about patient’s self-perception of feeling of fear during the procedure on a 100-point scale. Thereafter, patients were placed lying in a quiet environment with eyes closed but continued to feel the stress of the exposure. BAT test was determined through the subject’s performance in this specific height-related virtual environment. PET images, acquired in a lying position as recommended in standard practice, started 30 min after injection and ended 15 min later. Reconstruction used the ordered-subset expectation maximization algorithm with 5 iterations and 32 subsets and was corrected for attenuation using a CT transmission scan.

Whole-brain statistical analysis was performed at the voxel level using SPM8 software (Statistical Parametric Mapping, Wellcome Department of Cognitive Neurology, University College, London) [11]. PET images were spatially normalized onto the PET template of the Montreal National Institute (MNI) space [12], smoothed with a Gaussian filter (8 mm full-width at half-maximum), resulting in 2 × 2 × 2 mm voxel images. Proportional scaling was applied. SPM (T) maps were generated for correlation of metabolism with clinical scores before therapy using regression linear models and comparison before and after VRET using paired *t* tests (*p* voxel < 0.005, corrected for cluster volume, with age and gender as nuisance covariates). To evaluate metabolic connectivity from the previously identified metabolic clusters in the intergroup comparison, IRCA was performed as previously described [13], with the same nuisance covariates and threshold as previously detailed for the group SPM (T) map comparisons.

## Results

### Patients

Before VRET, 18 patients with acrophobia were assessed by a brain ^18^F-FDG PET scan sensitized by virtual exposure during the radiopharmaceutical administration. However, 9 patients did not complete VRET. The therapeutic program and its required availability were considered to be too heavy by these patients. Therefore, only 9 patients were tested again with sensitized PET after VRET. Significant improvements in BAT and SUD scores were noticed after VRET (paired *t* test *p* values < 0.01 and 0.02, respectively) (Table 1).

### Correlations between metabolism and clinical scores before VRET

**Fig. 2:**
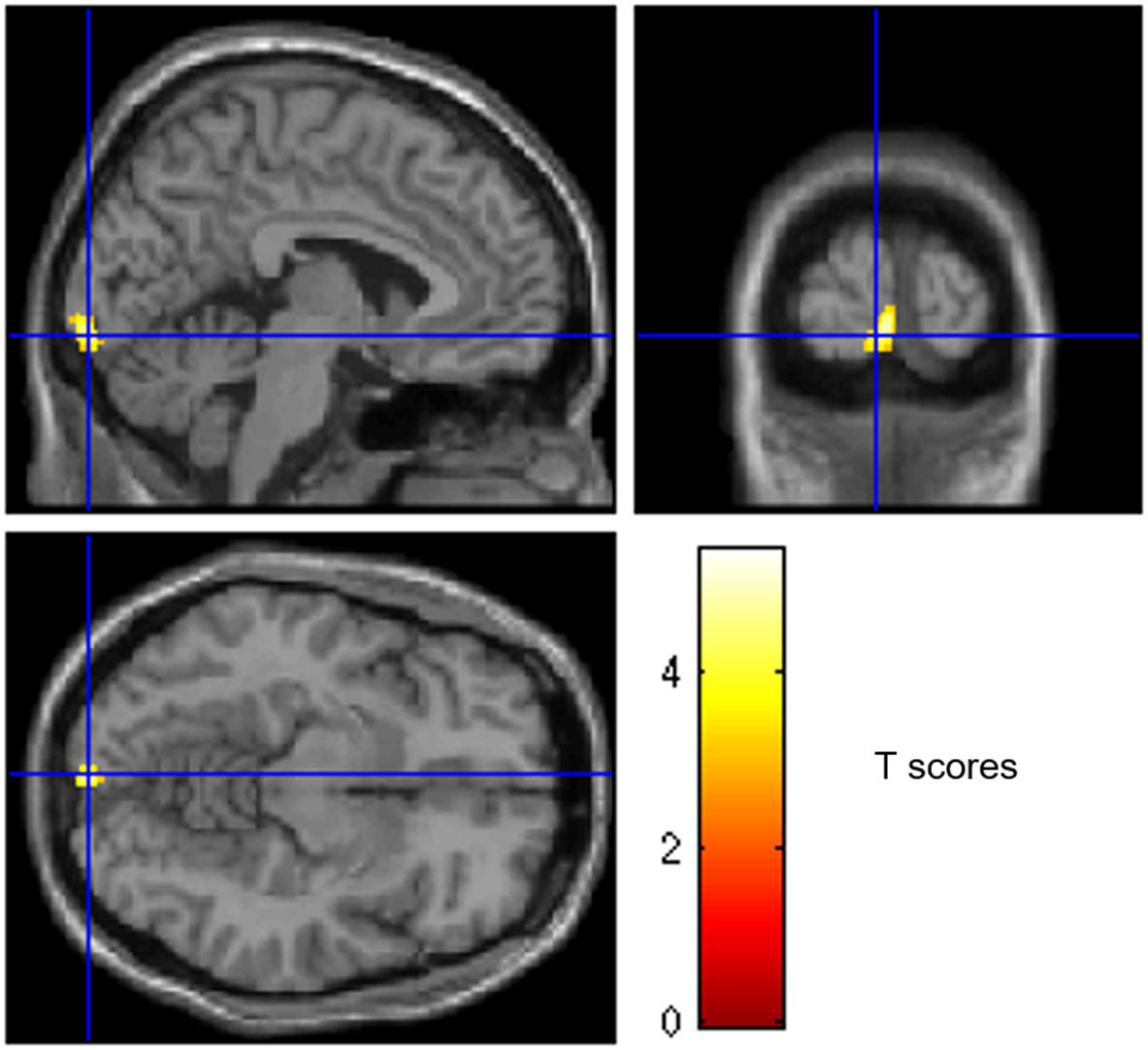
Anatomical localization of areas of increased metabolism in patients with acrophobia in relation to increased BAT score before treatment, projected onto sections of a standard SPM8 MRI template. Before treatment, a positive correlation was found between the BAT score and metabolism of the left occipital areas (BA17-18) (*p* < 0.005, *k* > 90). *L*: left, *R*: right

A positive correlation (Spearman correlation coefficient of 0.68) was found between BAT scores and the metabolism of the left occipital areas (BA17-18; *p* voxel < 0.005, corrected for cluster volume; Fig. 2). No negative correlation with the BAT score was found, and no correlation with SUD score was found.

### Changes in metabolism after VRET

**Fig. 3:**
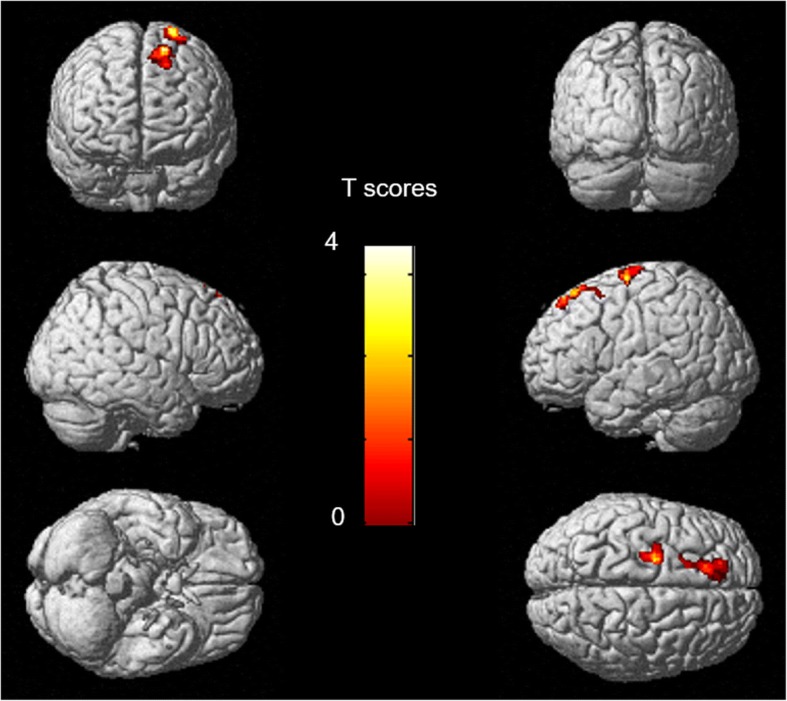
Anatomical localization of areas of increased metabolism in patients with acrophobia after VRET (*p* < 0.005, *k* > 178), projected onto 3D volume rendering. After VRET, patients showed increased metabolism in left superior frontal gyri (BA6-8) and in the left precentral gyrus (BA8). *L*: left, *R*: right

The comparison among the 9 patients who were assessed with ^18^F-FDG PET before and after VRET showed that patients with acrophobia had increased metabolism in the left superior frontal gyrus (BA6-8) and in the left precentral gyrus (BA8) after therapy (*p* voxel < 0.005, corrected for cluster volume; Fig. 3). No decreased metabolism was found.

### Changes in metabolic connectivity after VRET

**Fig. 4:**
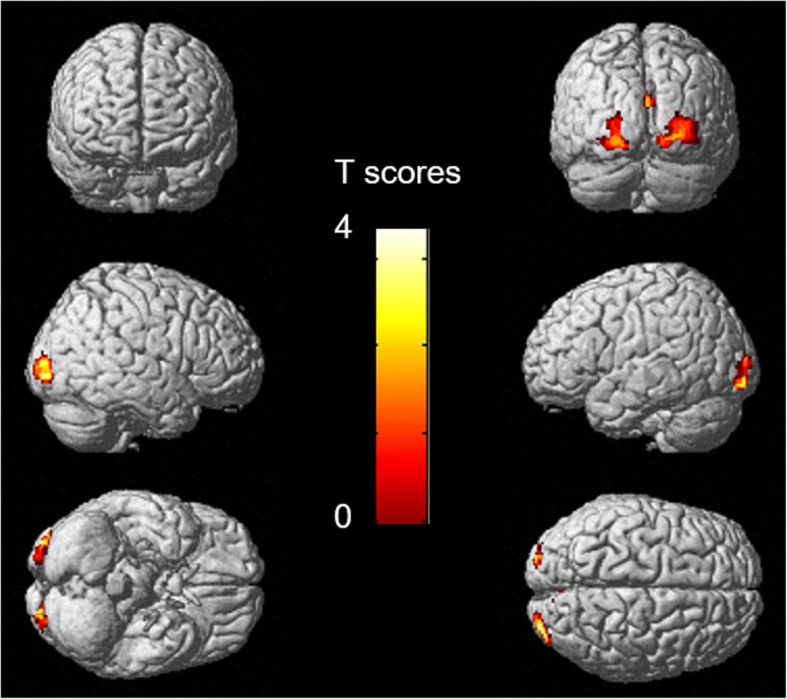
Anatomical localization of areas of increased left precentral gyrus connectivity in patients with acrophobia after VRET (*p* < 0.005, *k* > 84) projected onto 3D volume rendering. After the VRET, patients showed increased connectivity between the left precentral gyrus and bilateral occipital areas (BA17-18-19). *L*: left, *R*: right

Patients with acrophobia showed increased connectivity between the left precentral gyrus and the bilateral occipital areas (BA17-18-19) after VRET (Fig. 4), with Spearman coefficients of correlation between both areas of 0.24 (*p* = 0.34) before treatment and 0.73 (*p* = 0.03) after treatment. No decreased connectivity was found, as well as no changes in connectivity of the left superior frontal gyri.

No correlation between metabolism of the aforementioned clusters and BAT and SUD scores after VRET was noticed.

## Discussion

This pilot PET study showed that in patients with acrophobia before therapy, a lower BAT score was associated with a lower metabolism in occipital areas. In these patients, VRET led to increased metabolism in left superior frontal gyri and left precentral gyrus, areas commonly involved in motricity in relation to afferent sensorial stimuli, concomitant with clinical recovery. Interestingly, increased connectivity between the left precentral gyrus and occipital areas has been noticed after treatment, suggesting that clinical improvement induced by VRET could be the result of an increased weight of connectivity inside the visual-motor system network.

One of the most novel aspects of the current study is the use of PET imaging sensitized by virtual exposure for the first time, in order to measure the VRET response. Indeed, unlike functional magnetic resonance imaging (fMRI), PET imaging provides the unique opportunity to study functional imaging during virtual exposure by dissociating the time of the radiopharmaceutical injection from the acquisition of scans. Therefore, this procedure could be extended to different virtual environments or other mental disorders treated by VRET.

The present study confirms the effectiveness of VRET in the treatment of acrophobia [5, 6, 14] since the clinical scores of BAT and SUD were significantly improved after treatment. Interestingly, after VRET, an increase in metabolism was observed in frontal premotor areas (right BA6-8), especially those involved in oculomotor coordination (BA8), and these results were obtained during virtual exposure. These changes may account for the increased ability to cope with fear as observed in the self-report questionnaire. In fact, similar to motion sickness, vertigo with heights is due to a conflict between the vision, somatosensory and vestibular systems [15]. This is in accordance with our results showing on the one hand a positive correlation between metabolism of occipital areas and BAT score before therapy and, on the other hand, the improvement of metabolism in motor areas, especially in those concerning oculo-motor function after VRET. In fact, during the eight therapeutic VR sessions in the CAVE, the subject was asked to develop his/her ability to move in a virtual environment confronted with intense phobia cues due to his/her fear of heights. The patient has thus to develop his/her motor functions during these sessions and especially to increase the relationships between visual and motor systems, which is underlined by our PET results. A motor bias due to the study design appears unlikely since paired test was used to compare patients before and after treatment through the same motor paradigm, and premotor and not primary motor cortices were identified.

The increase in connectivity with areas involved in vision strengthens the hypothesis that the effectiveness of VRET is related to visual-motor system rehabilitation. Thus, people with an increased dependence on visual field information are characterized as less physically stable and more reliant on visual cues for controlling body stabilization [16–18]. This increased reliance on visual cues may be a vulnerability factor that promotes disequilibrium during certain situations of daily living, such as moving visual fields and heights, and thus may predispose to the development of acrophobia [19].

This pilot study nevertheless has some limitations. First, the main limitation is a low number of patients who followed this study to its full completion (*n* = 9). Second, a healthy control group explored with sensitized ^18^F-FDG PET is lacking, as well as a patient group not performing VRET.

## Conclusions

In conclusion, this pilot study highlights the exciting opportunity to use PET imaging to investigate brain metabolism during virtual exposure and provides additional information on the pathophysiology of acrophobia in the visual system. VRET improves metabolism in oculo-motor functional areas and enhances connectivity inside the visual-motor system, which is probably the key to the effectiveness of this treatment. Further studies involving a larger number of patients with control groups are needed to validate these preliminary findings.

## Abbreviations

BA: Brodmann area
BAT: Behavioural avoidance test
DSM: Diagnostic and statistical manual of mental disorders
HMD: Head-mounted display
IRCA: Inter-regional correlation analysis
SPM: Statistical Parametric Mapping
SUD: Subjective unit of discomfort
VRET: Virtual reality exposure therapy
